# Evaluation of Information Generated by ChatGPT on Preventing Peripheral Venous Catheter‐Related Infections

**DOI:** 10.1002/nop2.70441

**Published:** 2026-02-28

**Authors:** Seda Pehlivan, Derya Akça Doğan, Öznur Erbay Dallı

**Affiliations:** ^1^ Department of Internal Medicine Nursing, Faculty of Health Sciences Bursa Uludag University Bursa Turkey

**Keywords:** artificial intelligence, catheter‐related infections, knowledge, natural language processing, peripheral catheterisation

## Abstract

**Aim:**

To evaluate the accuracy and completeness of information generated by ChatGPT models in preventing peripheral intravenous catheter‐related infections.

**Background:**

Peripheral intravenous catheters are vital for administering medication and fluids but often cause complications like life‐threatening infections. These issues increase healthcare costs and patient discomfort. Nurses are crucial in managing these catheters, yet studies show they often lack knowledge and adherence to best practices.

**Method:**

This descriptive study utilised a 10‐question Information Form for Preventing Peripheral Venous Catheter‐Related Infections. By presenting the form to ChatGPT models (GPT‐3.5, GPT‐4, and GPT‐4o), it was requested that each multiple‐choice question be answered and a brief explanation provided as to why that option was correct. Responses were evaluated for correctness (0–10), accuracy (1–5), and completeness (1–3) using Likert scales.

**Results:**

GPT‐3.5 and GPT‐4o each scored 5 out of 10 on the Information Form for Preventing Peripheral Venous Catheter‐Related Infections, while GPT‐4 scored 3 out of 10. All models correctly answered questions on catheter replacement, selection, and dressing regimens (time of replacement) but struggled with hand hygiene, aseptic technique, and catheter dressing regimens (type of dressing). Accuracy scores averaged 3.9 for GPT‐3.5, 3.5 for GPT‐4, and 3.4 for GPT‐4o. Completeness scores averaged 1.8 for GPT‐3.5, 1.6 for GPT‐4, and 1.8 for GPT‐4o. There were no significant differences in accuracy and completeness scores between the models (*p* > 0.05).

**Conclusion:**

The findings highlight that although ChatGPT models can provide supportive information, their limitations in accuracy and completeness may pose risks for patient safety, particularly in critical domains such as aseptic technique and hand hygiene. It was emphasised that expert supervision is needed in the use of artificial intelligence tools to provide information in healthcare services.

**Impact:**

Future improvements in artificial intelligence models are necessary to enhance their effectiveness in medical applications. It is crucial to ensure that healthcare professionals are aware of the current limitations of artificial intelligence tools and continue to rely on expert knowledge and supervision in clinical settings.

**Patient or Public Contribution:**

No patient or public contribution.

## 1 Introduction

Peripheral intravenous catheters (PVCs), often the first invasive vascular access for many patients, are used about 2 billion times annually worldwide (Blanco‐Mavillard et al. 2023). Nurses play a crucial role in inserting, managing, and removing PVCs. These processes are part of the nurse's responsibilities, requiring adequate knowledge and practice. However, numerous studies have shown that nurses' knowledge and practices regarding the care of PVCs and adherence to evidence‐based guidelines are insufficient (August et al. 2019; Cicolini et al. 2014; Keogh et al. 2015; Raynak et al. 2020; Simonetti et al. 2019; Tosun et al. 2020).

Technology‐supported solutions can significantly contribute to nurses' education, enhancing their knowledge and skills. In the field of nursing education, Chat Generative Pre‐trained Transformer (ChatGPT) has emerged as a noteworthy artificial intelligence (AI) tool that deserves attention. As a generative AI chatbot, ChatGPT provides versatile information support by offering personalised and interactive assistance. However, before adopting and implementing ChatGPT as an information resource in nursing, it is crucial to understand its benefits and evaluate the associated risks (Abujaber et al. 2023). The data obtained from the studies to be conducted in this context is crucial for evaluating the usability of ChatGPT as a reliable educational information source for nursing educators, practitioners, and students. However, studies conducted on this subject are quite limited in terms of both number and quality (Kleib et al. 2024). Increasing the number and variety of studies in this field is important in order to make the most of ChatGPT's strengths and opportunities, while reducing the risks associated with its weaknesses and threats.

## 2 Background

PVCs are essential for administering medications and fluid therapies to nearly 80% of hospitalised patients (Massey et al. 2023). However, a significant portion of adult patients develop complications related to PVCs. These complications include phlebitis, extravasation, blockage, and life‐threatening infections (Indarwati et al. 2020; Kim et al. 2024; Marsh et al. 2018; Vinograd et al. 2018). When complications arise, a new catheter must be inserted to continue treatment. Re‐insertion attempts and early removal of PVCs increase hospital costs, including catheter expenditures and medical or nursing time (Alexandrou et al. 2015, 2018; Vinograd et al. 2018). The inability to establish and maintain peripheral intravenous access can delay diagnostic processes and subsequent medical treatments, potentially leading to patient discomfort, increased pain and anxiety, and a higher risk of preventable health issues (Marsh et al. 2018, 2020; Vinograd et al. 2018). When nurses responsible for PVC application and maintenance are equipped with evidence‐based knowledge and conduct clinical practices in accordance with guidelines, it will be possible to prevent all these adverse outcomes. However, studies have revealed that nurses' knowledge and practices in this area are inadequate.

Nurses, who are an important part of the healthcare system and provide uninterrupted service, have long faced serious challenges such as insufficient human resources and excessive workloads. It is reported that the global nursing shortage will reach 4.5 million by 2030, which will seriously affect the accessibility and quality of healthcare services. At the same time, factors such as the aging population and the increasing burden of chronic diseases, which are the result of changing demographics around the world, are accelerating this process. This growing need and these constraints have brought to the fore the possibility of utilising technological resources that can produce quick solutions in nursing education, practice, and teaching. In recent years, research into how generative AI can be used to increase nursing efficiency, optimise nursing care quality, and improve patient outcomes has become important (Peng et al. 2025).

Natural Language Processing (NLP) models have made significant progress in analysing textual data, providing personalised recommendations, and offering on‐demand support due to advances in deep learning techniques and large datasets over the past decade. Chat Generative Pre‐trained Transformer (ChatGPT) is an advanced NLP model, utilising 175 billion parameters, trained on extensive datasets to produce responses that resemble human interaction (Fuchs 2023; Gilson et al. 2023). ChatGPT is a trending AI tool developed by OpenAI. It was first released in November 2022 as GPT‐3.5, followed by the second version GPT‐4 in 2023, and the GPT‐4o version in 2024. Two months after its initial release, the number of monthly active users exceeded 100 million, reaching over 180.5 million users in 2024 (Gunawan et al. 2024; Miao and Ahn 2023). Even among users new to artificial intelligence, ChatGPT is quickly gaining acceptance due to its simple user interface. More importantly, it is often claimed that it is quite capable of answering a wide variety of questions like a knowledgeable human and is likely more knowledgeable than many human educators, especially on basic and intermediate topics across nearly every discipline (Miao and Ahn 2023). The potential impact of ChatGPT, which is developing, evolving, gaining acceptance, being used, and rapidly integrating into life at such a rapid pace, on nursing education and practice is a topic that warrants investigation. However, it is reported that the majority of the numerous publications found in the literature regarding the use of ChatGPT in nursing consist of editorials, commentaries, and essays. It is also emphasised that there is an urgent need for more empirical studies examining the impact of ChatGPT on nursing practice and research. Furthermore, although these tools can communicate in many languages besides English, it has been noted that their proficiency in each language depends on the quality and quantity of available educational data in that language, making it important to identify shortcomings in studies conducted in different countries and languages (Kleib et al. 2024).

Although these models can potentially address knowledge gaps in healthcare, they are only partially error‐free (Harrer 2023; Sallam 2023). The benefits of using ChatGPT in the healthcare field for diagnosis and information support, personalisation of care, decision‐making, and documentation are emphasised. However, studies related to nursing education and practice highlight that there are risks involved, such as providing incorrect information and references, and in some cases, experiencing “hallucinations or confusion” (Kleib et al. 2024). AI has revolutionary potential in both nursing education and practice, but this potential must be carefully managed to be fully realised (Le Lagadec et al. 2024). We aimed to evaluate the information generated by ChatGPT, which is increasingly being used for educational, practical, and informational purposes, regarding the prevention of PVC‐related infections, which are important in patient care and are performed millions of times daily by nurses. It is believed that the results of this study will provide information about ChatGPT's potential to serve as a reliable source for nurse educators, practitioners, and students.

## 3 The Study

### 3.1 Aim of Study

This study aimed to evaluate the information provided by ChatGPT and its references based on the information form for PVCs‐related infections.

## 4 Methods

### 4.1 Study Design

This study is a descriptive study that evaluates the information generated by ChatGPT regarding the prevention of peripheral venous catheter‐related infections. The “Information Form for Preventing Peripheral Venous Catheter‐Related Infections” was used to evaluate information aimed at preventing peripheral venous catheter‐related infections. The questions in the relevant form were asked to all three ChatGPT models, and the generated responses were analysed.

#### 4.1.1 Information Form for Preventing Peripheral Venous Catheter‐Related Infections

The form aims to quickly measure nurses' practice and knowledge levels in preventing PVC‐related infections (Cicolini et al. 2014). This form was developed by Cicolini et al. based on the Centers for Disease Control and Prevention (CDC) guidelines (O'Grady et al. 2011). Tosun et al. validated the form for face and content validity for our country (Tosun et al. 2020). The form contains 10 multiple‐choice questions, each with four options. The topics covered by these questions are: (1) PVC replacement; (2) hand hygiene; (3) aseptic technique; (4) selection of catheters; (5) catheter site dressing regimens (time of replacement); (6) catheter site dressing regimens (type of dressing); (7) skin preparation; (8) catheter site dressing regimens (antibiotic ointment); (9) replacement of administration sets (lipid emulsion infusions); and (10) replacement of administration sets (neither lipid emulsions nor blood product infusions). The answers provided for each question included one correct answer and three distractors. Correct answers are scored 1 point and incorrect answers 0 points, with a total score range of 0 to 10, where higher scores indicate increased knowledge (Cicolini et al. 2014).

### 4.2 Evaluation Process

#### 4.2.1 Stage 1: Collecting Model Responses

##### 4.2.1.1 Question Presentation

The questions in the form were evaluated on ChatGPT‐3.5, ChatGPT‐4, and ChatGPT‐4o models developed by Open Artificial Intelligence (OpenAI). The questions were asked to the models on May 26, 2024.

##### 4.2.1.2 Instruction

The model was instructed to act like a nursing graduate and respond based on the latest health research, best practices, and nursing topics. The command was: “Please act as a nursing graduate and respond based on the latest health research, best practices, and nursing topics.” The input instruction to the model was: “Please choose the correct answers to the following questions and provide a brief explanation for each of your choices. Limit your responses to a maximum of 5 sentences.” This instruction evaluated the model's ability to provide accurate information and logical reasoning while responding to a series of questions.

##### 4.2.1.3 Recording Responses

The model's responses and explanations were recorded. The response evaluation process was conducted independently by two researchers.

#### 4.2.2 Stage 2: Accuracy and Completeness Assessment

##### 4.2.2.1 Initial Scoring

Responses to multiple‐choice questions were first scored as correct or incorrect (1 or 0).

##### 4.2.2.2 Detailed Evaluation

Subsequently, regardless of whether the answers given to the multiple‐choice questions were correct or incorrect, the explanatory information generated for each question was evaluated in terms of accuracy and completeness. In the evaluation, the accuracy and completeness scale defined by Johnson et al. and used in previous studies, as shown in Table 1, was employed (Coskun, Yagiz, et al. 2023; Johnson et al. 2023). According to this evaluation by these two researchers, the accuracy score of the information generated by the models was rated between 1 and 5, and the completeness score was rated between 1 and 3.

**TABLE 1:** The accuracy and completeness assessment.

Accuracy assessment Completely incorrect More incorrect than correct Approximately equal amounts of correct and incorrect More correct than incorrect Completely correct
Completeness assessment Inadequate: The response addresses some aspects of the question but is missing significant parts. Adequate: The response contains sufficient details and basic information about the question. Comprehensive: The response covers all aspects of the question and offers more information or context than expected.

##### 4.2.2.3 Reconciliation

Following the evaluation of responses, in the event of a discrepancy in scoring between two researchers, the decision was made based on the scoring of the third researcher.

#### 4.2.3 Stage 3: Reference and Source Verification

After obtaining the answers, the model was asked to provide explicit references or sources for the information in its responses. The command was: “Can you provide the references for the information used in your above answers?” The references were evaluated and recorded by experts. To minimise memory retention bias, a new session was started for each question.

### 4.3 Data Analysis

The analyses were performed using SPSS version 22 (IBM Statistical Package for Social Sciences). Kruskal–Wallis test was used to compare the AI models based on their accuracy and completeness scores. A significance level of *p* < 0.05 was considered.

### 4.4 Ethical Considerations

As all responses were generated by a pre‐trained AI language model (ChatGPT) based on existing data, and no human or animal data was used in this study, ethical approval was not required.

## 5 Results

GPT‐3.5 and GPT‐4o each scored 5 points on the Information Form for Preventing Peripheral Venous Catheter‐Related Infections, while GPT‐4 scored 3 points. Therefore, GPT‐3.5, GPT‐4, and GPT‐4o achieved correct answers at 50%, 30%, and 50%, respectively. Correct and incorrect answers for each question are shown in Table 2.

**TABLE 2:** Responses to multiple‐choice knowledge questions according to models.

Questions	GPT‐3.5	GPT‐4	GPT‐4o
1. PVC replacement	1	1	1
2. Hand hygiene	0	0	0
3. Aseptic technique	0	0	0
4. Selection of catheters	1	1	1
5. Catheter site dressing regimens (time of replacement)	1	1	1
6. Catheter site dressing regimens (type of dressing)	0	0	0
7. Skin preparation	1	0	0
8. Catheter site dressing regimens (antibiotic ointment)	0	0	1
9. Replacement of administration sets (lipid emulsion infusions)	1	0	1
10. Replacement of administration sets (neither lipid emulsions and nor blood product infusions)	0	0	0
Total correct answers	5	3	5

*Note:* 0, Incorrect; 1, Correct.

All three models correctly answered questions related to PVC replacement, selection of catheters, and catheter site dressing regimens (time of replacement). Only GPT‐3.5 correctly answered the question on skin preparation, while only GPT‐4o correctly answered the question on catheter site dressing regimens (antibiotic ointment). GPT‐3.5 and GPT‐4o correctly answered the question about replacing administration sets (lipid emulsion infusions). However, all three models incorrectly answered questions on hand hygiene, aseptic technique, catheter site dressing regimens (type of dressing), and replacement of administration sets (neither lipid emulsions nor blood product infusions) (Table 2).

**FIGURE 1:**
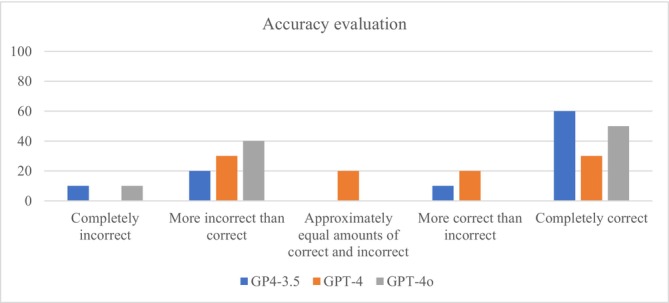
Accuracy evaluation of explanations provided by models.

**FIGURE 2:**
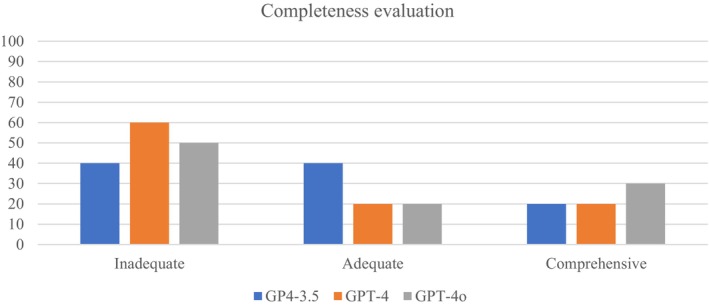
Completeness evaluation of explanations provided by models.

Responses, along with the provided explanations, were evaluated in detail using the accuracy and completeness scales. Table 3 compares the accuracy and completeness scores of GPT‐3.5, GPT‐4, and GPT‐4o models. The average accuracy scores were 3.9 for GPT‐3.5, 3.5 for GPT‐4, and 3.4 for GPT‐4o. However, statistical tests (χ^2^(2) = 0.605, *p* = 0.739) indicated this difference was insignificant. The accuracy of the models' explanations is shown in Figure 1. The average completeness scores were 1.8 for GPT‐3.5, 1.6 for GPT‐4, and 1.8 for GPT‐4o; this difference was also statistically insignificant (χ^2^(2) = 0.462, *p* = 0.794). The completeness of the models' explanations is shown in Figure 2.

**TABLE 3:** Distribution of accuracy and completeness evaluation of responses by models.

Questions	Accuracy score (1–5)				Completeness score (1–3)			
	GPT‐3.5	GPT‐4	GPT‐4o	Test statistic	GPT‐3.5	GPT‐4	GPT‐4o	Test statistic
1. PVC replacement	5	5	5	χ^2^(2) = 0.605 *p* = 0.739	2	3	3	χ^2^(2) = 0.462 *p* = 0.794
2. Hand hygiene	2	2	2		1	1	1	
3. Aseptic technique	1	2	2		1	1	1	
4. Selection of catheters	5	5	5		2	2	2	
5. Catheter site dressing regimens (time of replacement)	5	5	5		2	3	3	
6. Catheter site dressing regimens (type of dressing)	4	4	2		1	2	1	
7. Skin preparation	5	3	1		3	1	1	
8. Catheter site dressing regimens (antibiotic ointment)	5	4	5		3	1	2	
9. Replacement of administration sets (lipid emulsion infusions)	5	2	5		2	1	3	
10. Replacement of administration sets (neither lipid emulsions and nor blood product infusions)	2	3	2		1	1	1	
Mean	3.9	3.5	3.4		1.8	1.6	1.8	

Abbreviation: PVC, peripheral intravenous catheters.

When reviewing the references, it was found that GPT‐3.5 did not provide specific references, while GPT‐4 and GPT‐4o generally recommended guidelines published by health authorities and professional organisations (Table 4). The organisations included the American Hospital Association (AHA), the American Nurses Association (ANA), the Association for Professionals in Infection Control and Epidemiology (APIC), the Centers for Disease Control and Prevention (CDC), the Infusion Nurses Society (INS), the International Council of Nurses (ICN), the Society for Healthcare Epidemiology of America (SHEA), and the World Health Organization (WHO). Additionally, GPT‐4o recommended the Infectious Diseases Society of America (IDSA). Furthermore, GPT‐4o suggested articles, journals, and books as references for some questions; however, the recommended articles were found to be incorrect and inaccessible. It was observed that GPT‐4 recommended the CDC as a source for six questions, and GPT‐4o recommended it for nine questions.

**TABLE 4:** Distribution of reference recommendations by GPT‐4 and GPT‐4o for questions. Distribution of references provided for the information generated for questions, compared to GPT‐4 and GPT‐4o.

References	Questions									
	1	2	3	4	5	6	7	8	9	10
AHA	GPT‐4				GPT‐4		GPT‐4		GPT‐4	
ANA						GPT‐4				
APIC	GPT‐4				GPT‐4		GPT‐4			GPT‐4
CDC	GPT‐4o	Both	GPT‐4o		Both	GPT‐4o	Both	Both	Both	GPT‐4o
ICN						GPT‐4				
IDSA					GPT‐4o			GPT‐4o		
INS	GPT‐4o	GPT‐4	GPT‐4o	GPT‐4o		GPT‐4o	GPT‐4o	GPT‐4	Both	Both
SHEA	GPT‐4				GPT‐4			GPT‐4	GPT‐4	GPT‐4
WHO	GPT‐4o	Both	GPT‐4o				GPT‐4o	Both	GPT‐4o	GPT‐4o
Article		GPT‐4o		GPT‐4o						
Book				GPT‐4o						
Journal					GPT‐4o					

Abbreviations: AHA, American Hospital Association; ANA, American Nurses Association; APIC, Association for Professionals in Infection Control and Epidemiology; CDC, Centers for Disease Control and Prevention; ICN, International Council of Nurses; IDSA, Infectious Diseases Society of America; INS, Infusion Nurses Society; SHEA, Society for Healthcare Epidemiology of America; WHO, World Health Organization.

## 6 Discussion

This study revealed that the information and references generated by ChatGPT models on preventing peripheral venous catheter (PVC)‐related infections varied and demonstrated overall low performance. Although large language models (LLMs) are capable of producing impressive and human‐like outputs, their effectiveness in real‐world healthcare situations requiring advanced reasoning and procedural expertise is still under evaluation (Lievin et al. 2024). Our findings highlight that while AI models can serve as supportive tools, their limitations as independent sources of information in critical decision‐making processes must be acknowledged.

When compared with studies evaluating nurses' knowledge using the same form, the performance of the AI models in this study was lower. Cicolini et al. (2014) found a median score of 6 (IQR: 5–7) among nurses, Tosun et al. (2020) reported an average of 4.51 ± 1.67, and Simonetti et al. (2019) found a median of 6 (IQR: 5–7) among nursing students. In contrast, GPT‐3.5 and GPT‐4o each achieved 5 points, while GPT‐4 achieved only 3 points. This suggests that AI currently needs improvement in mimicking and replacing the complex nature of human learning and practice. An important conclusion from this comparison is that AI systems should not be used as completely independent sources of information in medical knowledge and practice; instead, they can be more effective when reviewed and guided by experienced healthcare professionals.

In this study, GPT‐4 underperformed compared to GPT‐3.5 and GPT‐4o in terms of accuracy, which may be linked to differences in response‐generation patterns and knowledge representation. Similar findings were reported by Abosi et al. (2024), who noted that while LLMs performed reasonably well on general infection prevention inquiries, their performance declined for context‐specific tasks such as transmission‐based precautions that require precise procedural knowledge. He consistently low scores of all models on questions regarding aseptic technique and hand hygiene in our study further support this observation, as these domains demand strict adherence to clinical guidelines where even small variations in concentration, duration, or sequencing can determine correctness. Prior reviews have emphasised that LLMs, although capable of producing human‐like outputs, often struggle with accuracy and completeness when confronted with tasks requiring procedural precision (Busch et al. 2025). In addition, recent evidence indicates that large language models are vulnerable to *knowledge drift* and may not remain concordant with evolving clinical guidelines, leading to outdated or inconsistent responses over time (Nwachukwu et al. 2025). Taken together, these results suggest that while LLMs can serve as supportive tools in infection prevention education, their reliability in areas requiring context‐dependent and protocol‐specific knowledge remains limited. Importantly, while all 10 items in the Information Form are grounded in CDC guidelines and therefore relevant to patient safety, errors in domains such as aseptic technique and hand hygiene may have greater safety implications than others, underscoring the need to interpret AI performance not only by overall accuracy but also by its potential impact on patient outcomes.

Furthermore, in line with previous studies, questions 1, 5, and 9 in the form had the highest correct response rates (Cicolini et al. 2014; Simonetti et al. 2019; Tosun et al. 2020). In our study, GPT‐3.5 and GPT‐4o correctly answered all three, while GPT‐4 answered two. This suggests that both nurses and AI models perceive these questions as more straightforward and easily interpretable, reflecting their well‐designed structure. By contrast, more complex procedural areas—such as hand hygiene, aseptic technique, and administration set replacement—remain major challenges for AI models, underscoring the need for improvement in these domains. Investigating information generated by ChatGPT in various fields has shown different results. For example, Coşkun et al. reported that GPT‐4 was superior in accuracy and comprehensiveness in providing information about methotrexate use (Coskun, Yagiz, et al. 2023). Antaki et al. found promising performance by ChatGPT in ophthalmology (Antaki et al. 2023), while Yeo et al. highlighted some limitations in diagnosis and preventive medicine despite extensive knowledge in answering questions related to cirrhosis and hepatocellular carcinoma (Yeo et al. 2023). Johnson et al. noted that ChatGPT generally provided accurate and complete responses to medical questions but had some significant limitations (Johnson et al. 2023). Lastly, Coşkun et al. found that ChatGPT had limited performance in educating patients about prostate cancer, which could lead to misunderstandings and errors (Coskun, Ocakoglu, et al. 2023). These results reveal both the potential and current limitations of ChatGPT in providing medical information across different fields.

The performance of GPT‐4 and GPT‐4o in providing recommended references demonstrated the capacity to offer reliable sources for specific situations. The Information Form for Preventing Peripheral Venous Catheter‐Related Infections was developed based on CDC guidelines (Cicolini et al. 2014; O'Grady et al. 2011). It was observed that GPT‐4 recommended the CDC as a reference for six questions and GPT‐4o for nine questions. This result suggests that the models could effectively direct users to accurate information sources. However, using these tools should always be under the supervision of experts, and the accuracy of the information provided by language models should always be verified. ChatGPT AI models can be helpful as supportive tools in healthcare but should not be used as independent sources of information in critical decision‐making processes. This study is an essential step in better understanding the potential and limitations of language models in healthcare applications.

### 6.1 Strengths and Limitations

This study has several strengths. Firstly, it provided a comprehensive assessment of the information generated by three versions of ChatGPT (GPT‐3.5, GPT‐4, and GPT‐4o) regarding the prevention of peripheral intravenous catheter‐related infections. The use of a structured 10‐question Information Form ensured a systematic approach to the evaluation. Secondly, by comparing the performance of AI models against previously established nurse knowledge benchmarks, the study offered valuable insights into the relative capabilities of these models. Lastly, the objective scoring using Likert scales for accuracy and completeness provided a quantifiable measure of the AI responses, contributing to the robustness of the evaluation.

However, the study also had limitations. The scope was limited as it focused solely on the prevention of peripheral intravenous catheter‐related infections, which may not be generalisable to other areas of healthcare. Additionally, the study did not include a large‐scale validation with actual clinical outcomes, which would have further strengthened the findings. Finally, the reliance on pre‐defined questions may not fully capture the AI models' potential in more dynamic and varied clinical scenarios.

### 6.2 Recommendations and Implications

#### 6.2.1 For Further Research

Further research should aim to expand the scope of evaluation to include a broader range of clinical topics and scenarios, particularly those relevant to nursing education and practice. Large‐scale validation studies involving actual clinical outcomes are essential to better understand the practical implications of using AI models like ChatGPT in healthcare settings. Comparative studies across languages and cultural contexts would also be valuable to assess the generalisability of model performance, with specific attention to how such tools can support nursing students and professionals in different healthcare systems.

#### 6.2.2 For Policy and Practice

The findings of this study have important implications for policy and practice in healthcare, especially in nursing education and clinical decision‐making. While AI models like ChatGPT can serve as supportive tools, they should not be relied upon as independent sources of information in critical decision‐making processes. Education and training for healthcare professionals, and specifically for nursing students, on the limitations and appropriate use of AI tools are crucial. By implementing these measures, the healthcare sector can effectively harness the potential of AI while safeguarding patient safety and care quality. Additionally, policies should be developed to guide the integration of AI into healthcare practices, ensuring that AI‐generated information is always reviewed and validated by experienced professionals, such as nurse educators and clinical mentors.

#### 6.2.3 For AI Developers

Our results also highlight practical directions for AI developers. Training datasets should be enriched with guideline‐based and procedural content, particularly for infection prevention practices where precise contextual knowledge is required. Fine‐tuning models with validated clinical protocols could reduce inaccuracies in stepwise tasks such as aseptic technique and hand hygiene. Furthermore, incorporating real‐time updating mechanisms would help mitigate knowledge drift and ensure alignment with evolving clinical guidelines. Beyond infection prevention, these measures are relevant for broader clinical domains such as medication safety, device management, and nursing interventions, where procedural accuracy is equally critical. To support this progress, developers should collaborate closely with nursing professionals and educators to establish standardised benchmarks for evaluating AI performance across diverse scenarios. Such interdisciplinary collaboration will be key to improving the reliability, safety, and educational value of large language models in healthcare and nursing practice.

## 7 Conclusion

This study evaluated the performance of ChatGPT models in preventing PVCs‐related infections and found that their accuracy and completeness were limited. While AI tools can provide supportive information and complement clinical education, particularly in nursing curricula, they should never be used as independent sources in critical decision‐making processes. Their outputs must always be guided and verified by expert supervision to ensure patient safety and educational quality. Future studies are needed to determine the suitability of different model versions for specific healthcare and nursing applications. Finally, strengthening interdisciplinary collaboration between healthcare professionals, nurse educators, and AI developers is essential to improve the applicability, reliability, and safe integration of AI in healthcare.

## Author Contributions

Seda Pehlivan, Derya Akça Doğan, Öznur Erbay Dallı made substantial contributions to conception and design, or acquisition of data, or analysis and interpretation of data. Seda Pehlivan, Derya Akça Doğan, Öznur Erbay Dallı involved in drafting the manuscript or revising it critically for important intellectual content. Seda Pehlivan, Derya Akça Doğan, Öznur Erbay Dallı given final approval of the version to be published. Each author should have participated sufficiently in the work to take public responsibility for appropriate portions of the content. Seda Pehlivan, Derya Akça Doğan, Öznur Erbay Dallı agreed to be accountable for all aspects of the work in ensuring that questions related to the accuracy or integrity of any part of the work are appropriately investigated and resolved.

## Funding

The authors have nothing to report.

## Disclosure

*Permission to Reproduce Material From Other Sources*: Not applicable. This study did not involve reproducing material from other sources.

*Statistics*: The authors have checked to make sure that our submission conforms as applicable to the Journal's statistical guidelines and Ö.E.D. is a member of our author team who is currently pursuing a master's degree in statistics.

## Ethics Statement

Ethical approval was not required for this study as it involved the evaluation of AI‐generated information and did not include human or animal subjects.

## Consent

The authors have nothing to report.

## Conflicts of Interest

The authors declare no conflicts of interest.

## Data Availability

The data that support the findings of this study are available from the corresponding author upon reasonable request.
